# Injectable Hydrogel-Based Combination Cancer Immunotherapy for Overcoming Localized Therapeutic Efficacy

**DOI:** 10.3390/pharmaceutics14091908

**Published:** 2022-09-08

**Authors:** Jeongrae Kim, Yongwhan Choi, Dong-Hwee Kim, Hong Yeol Yoon, Kwangmeyung Kim

**Affiliations:** 1 KU-KIST Graduate School of Converging Science and Technology, Korea University, 145 Anam-ro, Seonbuk-gu, Seoul 02841, Korea; 2 Medicinal Materials Research Center, Biomedical Research Division, Korea Institute of Science and Technology (KIST), 14 Gil 5, Hwarang-ro, Seongbuk-gu, Seoul 02792, Korea; 3 Noxpharm Co. 924B, 14 Gil 5, Hwarang-ro, Seongbuk-gu, Seoul 02792, Korea; 4 Graduate School of Pharmaceutical Sciences, College of Pharmacy, Ewha Womans University, Seoul 03760, Korea

**Keywords:** injectable hydrogel, cancer immunotherapy, drug delivery

## Abstract

Various immunotherapeutic agents that can elicit antitumor immune responses have recently been developed with the potential for improved efficacy in treating cancer. However, insufficient delivery efficiency at the tumor site, along with severe side effects after systemic administration of these anticancer agents, have hindered their therapeutic application in cancer immunotherapy. Hydrogels that can be directly injected into tumor sites have been developed to help modulate or elicit antitumor responses. Based on the biocompatibility, degradability, and controllable mechanochemical properties of these injectable hydrogels, various types of immunotherapeutic agents, such as hydrophobic anticancer drugs, cytokines, antigens, and adjuvants, have been easily and effectively encapsulated, resulting in the successful elicitation of antitumor immune responses and the retention of long-term immunotherapeutic efficacy following administration. This review summarizes recent advances in combination immunotherapy involving injectable hydrogel-based chemoimmunotherapy, photoimmunotherapy, and radioimmunotherapy. Finally, we briefly discuss the current limitations and future perspectives on injectable hydrogels for the effective combination immunotherapy of tumors.

## 1. Introduction

**Scheme 1:**
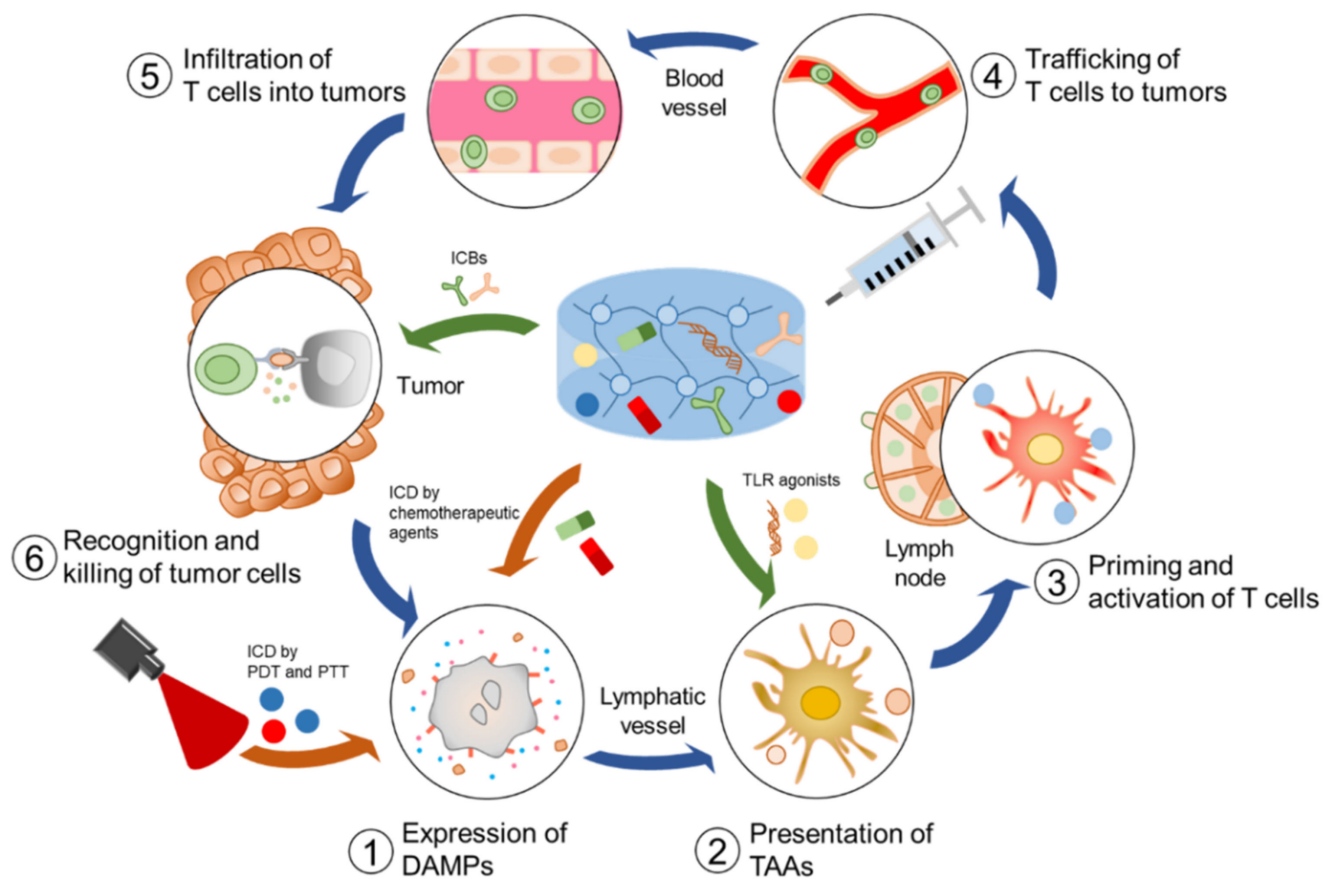
Schematic illustration of the cancer-immunity cycle based on the injectable hydrogels.

Localized delivery of anticancer therapeutics, which can provide sustained release of cytotoxic therapeutics at target sites, reduces the toxicity in normal tissues resulting from their systemic circulation [1,2]. The efficacy of localized therapy is significantly improved by increasing the local concentration of cytotoxic therapeutics at the target sites, offering the potential for overcoming limitations in systemic treatment, such as low solubility and delivery efficiency [3,4]. Thus, various drug delivery systems, including hydrogels [5,6,7], micelles [8], liposomes [9], and nano- or microparticles [10,11,12,13], have been utilized as local drug delivery reservoirs for the localized treatment of cancer. Among these, hydrogels that can form 3D network structures are widely used in biomedical fields, including tissue engineering, regenerative medicine, and drug delivery [14]. In particular, injectable hydrogels that undergo an in situ sol–gel transition in the body in response to chemical or external stimuli (e.g., pH, temperature, and light) after injection are widely utilized for local delivery owing to the easy loading of anticancer therapeutics [15]. By taking advantage of injectable hydrogels, single or multiple anticancer therapeutics, such as cytotoxic chemo drugs, cytokines, antibodies (Abs), phototherapeutics, and radiotherapeutics, can be implanted into target sites with minimal wounding via syringe injection [16,17]. Furthermore, controlled and sustained release of these therapeutics from the injectable hydrogels at target cancer sites enables effective treatment without inducing severe side effects in normal tissues. However, applications of typical local therapy using injectable hydrogels are often limited to early stage or isolated cancers because of their insufficient therapeutic effect against locoregional recurrence and metastatic cancers [18]. Recently, cancer immunotherapy has enabled the systemic treatment of various cancers, including recurrent and metastatic cancers, by activating and boosting anticancer immune responses to eliminate malignant cells, providing a breakthrough in overcoming the limitations of typical localized cancer therapy [19,20,21]. Despite advantages in the systemic treatment of cancers, high dosages or repeated local injections of cancer therapeutics to elicit anticancer immune responses are often limited in their therapeutic efficacy because of severe side effects [22]. Therefore, designing smart drug delivery systems that can provide sustained and controlled release of various types of cancer therapeutics has received attention for improving therapeutic efficacy while minimizing potential side effects [23]. From this perspective, injectable hydrogels can be used as efficient local delivery platforms to significantly improve the activation and boost the efficiency of anticancer immune responses to cancer immunotherapy [24]. Furthermore, injectable hydrogels can easily and efficiently implant one or more types of anticancer therapeutics, significantly enhancing therapeutic efficacy when used in combination with other conventional cancer therapies, such as chemotherapy, phototherapy, and radiotherapy [25,26,27,28]. Locally injected therapeutic agents that can induce immunogenic cell death (ICD) via specific therapeutic regimes, including photodynamic therapy (PDT) and photothermal therapy (PTT), generate damage-associated molecular patterns (DAMPs) in cancer cells. Then, tumor-associated antigens (TAAs) can be phagocytosed by antigen-presenting cells (APCs), resulting in the spontaneous activation of innate immune responses against cancer cells. Moreover, combined with adjuvants or immune modulators, such as toll-like receptor (TLR) agonists and immune checkpoint blockades (ICBs), these agents can boost the cancer-immunity cycle, leading to the establishment of potent T cell-mediated adaptive immunity (Scheme 1).

This review is organized as follows: Section 2 introduces injectable hydrogel-based immunotherapeutic agent delivery for activating and modulating anticancer immune responses, and Section 3 describes injectable hydrogel-based combination cancer immunotherapy, including chemoimmunotherapy, photoimmunotherapy, and radioimmunotherapy. Finally, in Section 4, we discuss the limitations and future perspectives on injectable hydrogels for clinical translation in cancer immunotherapy.

## 2. Injectable Hydrogels for Delivery of Immunotherapeutic Agents

Injectable hydrogels are gels that are mainly formed via chemical crosslinking or physical crosslinking after injection into the body [29,30]. Using injectable hydrogels, various types of therapeutics, including chemo drugs, Abs, proteins, and nucleic acids, can be readily packed during the gelation processes, as summarized in Table 1 [31,32]. Furthermore, these therapeutics can be sustainably released from the hydrogels into the tumor site, maintaining a relatively high drug concentration with less systemic toxicity. Various chemical reactions, such as Schiff’s base reactions, the Michael-type addition reaction, the Diels–Alder reaction, photochemical reactions, and disulfide formation, are widely utilized for chemical crosslinking, resulting in the formation of three-dimensional (3D) network structures via intermolecular covalent bond formation [33]. Chemically crosslinked hydrogels typically have a relatively lower critical gelation concentration, higher mechanical properties, and slow degradation compared to physically crosslinked hydrogels [34]. Various polymers have been used to form hydrogels by chemical crosslinking. Thomas et al. developed the F127/PEG hydrogel, composed of polyethylene glycol (PEG) and chemically conjugated with thermosensitive Pluronic^®^ F127 and branched PEI via thiolation [35]. The storage modulus of this hydrogel increases with its concentration. Specifically, the storage moduli of the 5.7% (*w*/*v*) and 2.7% (*w*/*v*) polymer weight hydrogels were about 1700 and 200 Pa, respectively. The anti-CTLA-4 antibody could be loaded into the F127/PEG hydrogel, and the hydrogel exhibited sustained release of the anti-CTLA-4 antibody, both in vitro and in vivo. Kurisawa et al. developed a biodegradable tyramine (Tyr)-conjugated hyaluronic acid (HA) hydrogel that incorporated interferon-α2a (IFN-α2a) for liver cancer therapy [36]. The physicochemical properties of the HA-Tyr hydrogel, such as the stiffness and gelation rates, could be determined by the extent of the oxidative coupling reaction catalyzed by horseradish peroxidase (HRP) and hydrogen peroxide (H_2_O_2_) during its formation. The storage modulus was 898 Pa for HA-Tyr-soft-IFN hydrogel and 3028 Pa for HA-Tyr-stiff-IFN hydrogel. The in vitro release profiles of IFN-α2a from HA-Tyr-soft-IFN hydrogel and HA-Tyr-stiff-IFN hydrogel were 78.5 and 46%, respectively, at 8 h. In vivo results showed that IFN-α2a released into the target tissues from the hydrogel made HAK-1B tumor cells more apoptotic and less proliferative; on the other hand, antitumor efficacy in the cytokine-treated group without a hydrogel was limited. Interestingly, better therapeutic outcomes resulted from the application of the soft hydrogel than from the stiff hydrogel, including a decrease in the incidence of angiogenesis owing to higher protein release and therefore, higher activity. Despite the benefits of using chemically crosslinked hydrogels for the local delivery of immunotherapeutics, potentially immunosuppressive reactions, including inflammation caused by crosslinkers, catalysts, organic solvents, etc., should be addressed for improving therapeutic efficacy after injection [37]. Another method for hydrogel formation is physical crosslinking via intermolecular interactions, including hydrophobic, electrostatic, host–guest, and hydrogen bonding interactions, wherein gelation processes can be reversed [38]. Hydrogels that are physically crosslinked can reduce the harmful effects on immunotherapeutics and antitumor immune responses in their application, since the physical sol–gel transition behavior is not dependent on catalysts and does not generate byproducts [39]. Chen et al. developed vitamin C amphiphile self-assembled nanofiber hydrogels (VitC hydrogel) for cancer immunotherapy [40]. Dodecyl carbon chain-conjugated VitC, with a hydrophilic head and hydrophobic tail, forms a nanofiber hydrogel via physical crosslinking. This hydrogel exhibited sustained in vitro and in vivo release of VitC for more than 1 week. Furthermore, SA@VitC hydrogel, additionally encapsulated with a stimulator of interferon genes (STING) agonist-4 (SA), enhanced antitumor immunity and showed prolonged survival up to 100% for 4 weeks. Although various studies have demonstrated that injectable hydrogels are advantageous due to their effective delivery and improved therapeutic efficiency by local delivery with sustained release of single or multiple anticancer therapeutics, locoregional recurrence and metastasis of cancers are lingering challenges in hydrogel-based cancer therapy [41]. Therefore, systemic therapeutic approaches are required to increase the therapeutic efficacy of injectable hydrogel-based therapeutics representative of localized therapy.

## 3. Injectable Hydrogels for Combination Cancer Immunotherapy

Among the conventional cancer therapies, chemotherapy, radiotherapy, and phototherapy can induce the ICD of cancer cells, activating anticancer immune responses [55]. Based on the anticancer immune responses, malignant cells or their phenotypes and functions can be eliminated or inhibited in the body [56]. However, immunosuppressive tumor microenvironments (TMEs) and multiple mechanisms in cancer cells, such as defects in antigen presentation and the regulation of regulatory signal pathways, enable the escape from immune surveillance, reducing anticancer immune responses and therapeutic efficacy [57]. Thus, a combination of therapeutic strategies that can overcome limitations in the various antitumor immune responses is needed to build strong and effective therapeutic alliances in cancer immunotherapy. In this regard, ICBs and various inhibitors such as indoleamine 2,3-dioxygenase (IDO), interleukin-10 (IL-10), and transforming growth factor-β (TGF-β) are widely used for combination cancer immunotherapy [58,59,60]. As described above, one of the main advantages of injectable hydrogels is that they can encapsulate various types of therapeutics and facilitate their sustained release. As a result, the application of a combination of conventional cancer therapy and immunotherapeutics using injectable hydrogels can result in improved therapeutic outcomes. In this section, we summarize the advantages of injectable hydrogel-based combination cancer immunotherapy and provide typical examples in each section.

### 3.1. Combination Chemoimmunotherapy

**Figure 1:**
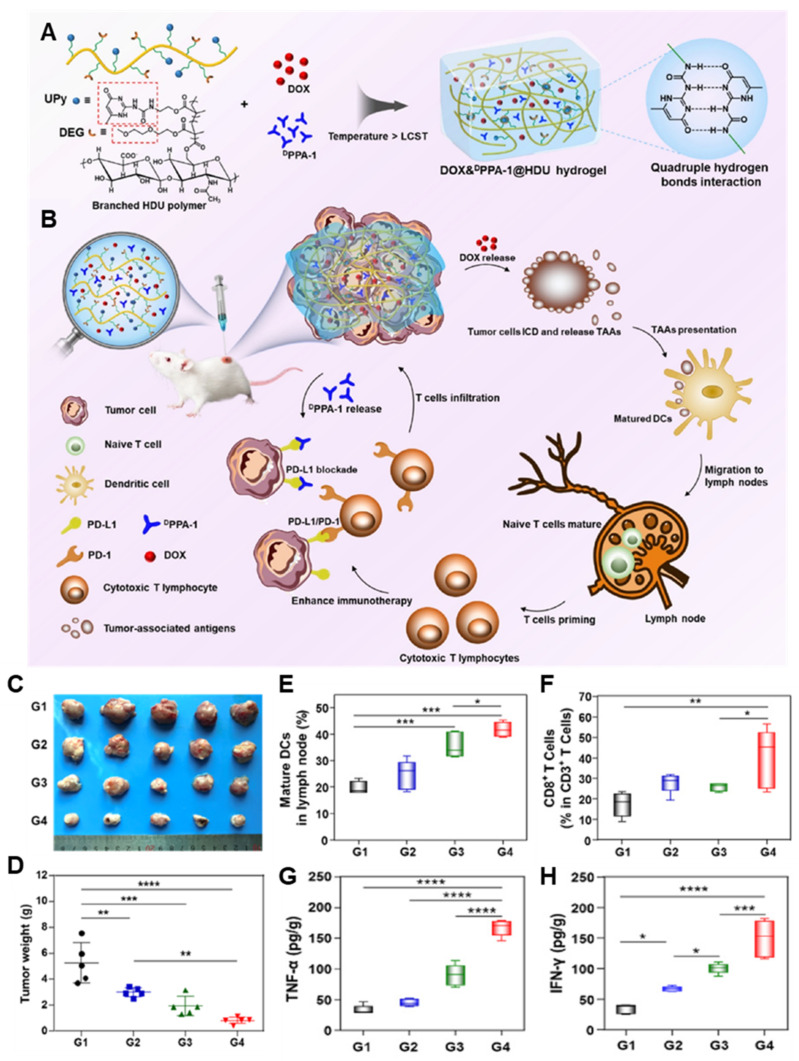
Injectable thermoresponsive hydrogel with ^D^PPA-1 and DOX for combination chemoimmunotherapy. (**A**) Schematic illustration of thermoresponsive hydrogel with DOX and ^D^PPA-1. (**B**) Schematic illustration of DOX and ^D^PPA-1@HDU hydrogel eliciting antitumor immune responses. (**C**) Photographs of primary tumors after treatments. (**D**) Tumor weight at the end of treatment. Populations of (**E**) mature DCs in LNs and (**F**) cytotoxic T cells in tumors analyzed using flow cytometry. Quantification of secreted amounts of (**G**) TNF-α and (**H**) IFN-γ in the tumors. * *p* < 0.05, ** *p* < 0.01, *** *p* < 0.001, **** *p* < 0.0001, one-way ANOVA for indicated comparison. Figures are reproduced from reference with permission [44] from the American Chemical Society.

Chemotherapy is a cancer treatment whereby cancer cells are directly killed using various chemicals, such as chemo drugs [61]. However, systemic administration of these chemo drugs results in fast clearance and off-target side effects, such as neurotoxicity, myelosuppression, and liver dysfunction, which often limit the therapeutic efficacy. Injectable hydrogels that locally deliver cytotoxic chemo drugs may provide more advanced approaches for durable and effective treatment than typical chemotherapy [62]. Recent studies have shown that some chemo drugs, including members of the anthracycline family, oxaliplatin, docetaxel, paclitaxel, and mitoxantrone, can trigger ICD in cancer cells, activating adaptive immunity by generating DAMPs and cytokines [63,64]. For example, Yang et al. developed a peptide nanofiber-based hydrogel loaded with a chemotherapy drug, doxorubicin (DOX), and melittin to activate an immunosuppressive TME [42]. DOX is an effective antitumor cytotoxic chemotherapy drug, as well as a representative ICD inducer, causing cancer cells to undergo apoptosis. Melittin is known as a cationic polypeptide anticancer agent that has the potential to deplete M2-like tumor-associated macrophages (TAMs). However, the hemolysis effect of melittin often limits its in vivo application. To overcome this limitation and enhance therapeutic efficacy, a DOX- and melittin-loaded hydrogel system (MRD hydrogel) was developed by conjugating amphiphilic synthetic RADA_32_ and cationic melittin peptides through the GG linker (sequence: RADARADARADARADARADARADARADARADA-GG-GIGAVLKVLTTGLPALISWIKRKRQQ-NH_2_). The MRD hydrogel formed self-assembled networks of interweaving nanofibers, releasing DOX and melittin into tumor tissue in a sustained manner. In all, 40.5% of MRD hydrogel-treated B16-F10 cells showed a necrotic cell population, which was 5.1- and 6.1-fold higher than that of cells treated with a combination of RD hydrogel and DOX. Furthermore, MRD hydrogel-treated B16-F10 cells were arrested in the G2/M phase and showed a reduction in the S phase, inhibiting the cell cycle. Moreover, the primary tumor growth was inhibited by more than 95%, owing to the sustained release of drugs in the TME. As a result of the antitumor immunity, dendritic cells (DCs), as well as T cells, were activated, and natural killer (NK) cells were recruited. Furthermore, the MRD hydrogel induced effective depletion of immunosuppressive macrophages via the sustained release of melittin from the hydrogel. Analysis of the complete response (CR) and the promising result of tumor rechallenge confirmed that strong immunity and the long-term memory effect were well established by the MRD hydrogel. Another approach to enhancing anticancer immune responses is to improve the activation and maturation of DCs in the tumor tissue [65]. DCs are representative APCs that can activate cytotoxic T cells [66,67]. Differentiation of immature DCs induced by maturation signals, including DAMPs, pathogen-associated molecular patterns (PAMPs), and cytokines, enables them to fully participate in the immune response, including in priming naïve T cells [68]. However, tumor-associated DCs are often functionally suppressed, requiring an adjuvant to facilitate DC activation and maturation [69]. Zhang et al. developed a DOX- and imiquimod (R837)-loaded injectable hydrogel that was composed of four-arm polyethylene glycol thiol (PEGSH) and poly(ethylene glycol) diacrylate (PEGDA) for the synergistic therapy of melanoma [43]. The hydrogel was formed within 5 min via a Michael addition reaction between PEGSH and PEGDA. The locally injected hydrogel was gradually degraded for 2 weeks in vivo. Owing to the DOX and R837 released from this hydrogel, apoptotic in addition to non-apoptotic cell death were induced. The DOX and R837 co-delivering hydrogel effectively inhibited in vivo tumor growth and metastatic progression through DOX-based ICD, with R837-based immune response amplification through DC maturation, M1 macrophage activation, tumor necrosis factor-α (TNF-α), and IFN-γ secretion. On the other hand, immunosuppressive responses including M2 macrophage infiltration and IL-10 secretion were alleviated, demonstrating that the hydrogel-based continuous release of DOX and R837 can provide an effective cancer treatment strategy. Immune checkpoint inhibitors that can target CTLA-4 and the programmed cell death 1 (PD-1)/programmed cell death ligand 1 (PD-L1) axis induce significantly enhanced therapeutic responses in cancer immunotherapy [70,71]. In combination with cytotoxic chemo drugs that directly act on cancer cells, immune checkpoint inhibitors elicit synergistic anticancer immune responses by interfering with co-inhibitory T-cell signaling [72]. Yang et al. developed a DOX and PD-L1 agonist peptide (^D^PPA-1)-loaded injectable hydrogel by polymerizing hyaluronic acid methacrylate (HAMA) with 2-(3-(6-methyl-4-oxo-1,4-dihydropyrimidin-2-yl)ureido)ethyl methacrylate (UPyMA) and diethylene glycol methacrylate (DEGMA), forming thermosensitive hydrogels (HDU hydrogels) [44]. The HDU hydrogel was rapidly formed by hydrophobic interactions and hydrogen bonding at 37 °C, and it then gradually released incorporated DOX and ^D^PPA-1 to provoke antitumor immunity, including potentiating T-cell immunity (Figure 1A). DOX-induced ICD activated innate immune responses through TAA presentation by a thermoresponsive hydrogel containing DOX and ^D^PPA-1. Subsequently, primed cytotoxic T cells elicited strong antitumor immune responses against the target tumor incapacitated by the PD-L1 blockade in a CT26 colon cancer mouse model (Figure 1B). Tumor growth was significantly inhibited following treatment using the DOX and ^D^PPA-1@HDU gel compared with the DOX- or ^D^PPA-1-loaded hydrogel (Figure 1C,D). The populations of mature DC and cytotoxic T cells after the treatment with DOX and ^D^PPA-1@HDU hydrogel were 2.1- and 2.3-fold higher, respectively, than that for the blank HDU hydrogel (Figure 1E,F). The amounts of TNF-α and IFN-γ in the DOX and ^D^PPA-1@HDU hydrogel-treated group were significantly higher than those in other groups (Figure 1G,H).

**Figure 2:**
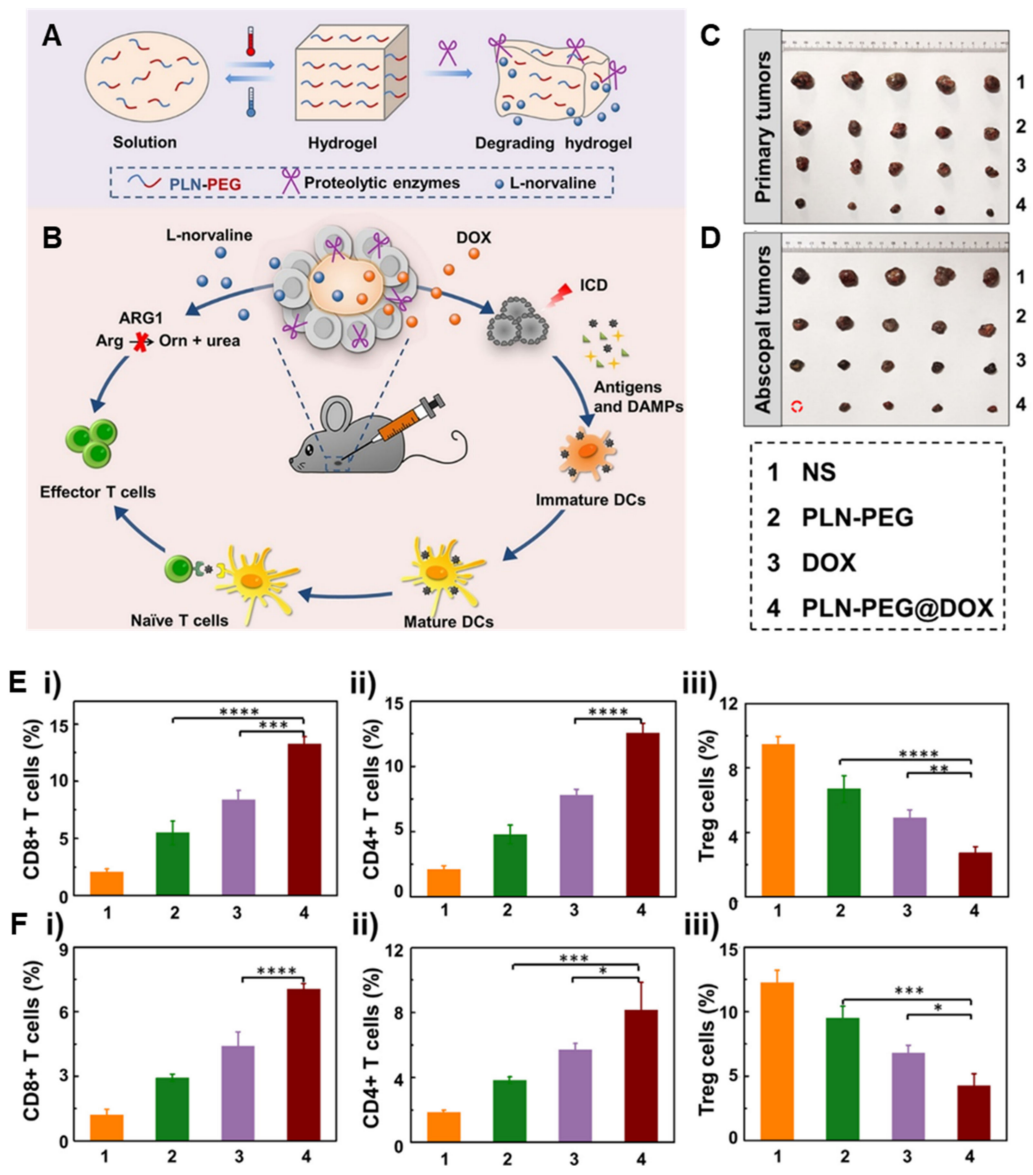
Injectable thermosensitive hydrogel with L-norvaline for combination chemoimmunotherapy. (**A**) Schematic illustration of thermosensitive hydrogel with L-norvaline. (**B**) Schematic illustration of L-norvaline-based immunomodulation based on blocking the ARG1 pathway. Photographs of (**C**) primary and (**D**) secondary tumors after treatments. (**E**) Populations of (**i**) CD8^+^, (**ii**) CD4^+^, and (**iii**) regulatory T cells in primary tumors. (**F**) Populations of (**i**) CD8^+^, (**ii**) CD4^+^, and (**iii**) regulatory T cells in secondary tumors. * *p* < 0.05, ** *p* < 0.01, *** *p* < 0.001, **** *p* < 0.0001, one-way ANOVA for indicated comparison. Figures are reproduced from [46], with permission from Elsevier.

CpG is known as one of the PAMPs and exhibits immunostimulatory activity through interacting with TLR9 expressed in immune cells such as DCs and B cells [73]. The resultant activated DCs and macrophages facilitate the production of cytokines, such as TNF-α and IL-12, leading to the enhancement of T cell-mediated antitumor immunity [74]. For example, chemoimmunotherapy using an immunostimulatory self-assembled CpG DNA hydrogel complex exhibited strong antiproliferative activity against colon26/Luc cells, with the in vitro activation of immune cells and effective in vivo tumor growth inhibition [75]. Lv et al. developed a DOX/CpG-loaded self-assembled nanoparticles (NPs)-exogenous DC incorporated cyclodextrin hydrogel system [45]. DOX elicits strong cytotoxicity, as well as triggers a series of immune responses by inducing the ICD of tumor cells. Additionally, CpG was introduced into this combination to augment antitumor immunity. To synthesize a pH-responsive prodrug, DOX was conjugated with polyethylenimine (PEI) via an amide bond using *cis*-aconitic anhydride (CA), forming PEI-CA-DOX. Owing to the positive charge of PEI-CA-DOX, CpG was successfully complexed by electrostatic interaction. Subsequently, DOX-CpG NPs and DCs were finally mixed with cyclodextrin to prepare the hydrogel system. Because of the pH sensitivity of the incorporated NP structure, the chemo drug side effect was reduced as a result of the controlled release. DOX was released relatively quickly under the acidic conditions, and sustained release of CpG supported the prolonged immune stimulation. Co-delivered exogenous DCs interacted with tumor antigens generated by DOX released from the hydrogel. The resultant antigen presentation promoted immunogenic immune responses, reprogramming the immunosuppressive TME (ITM) through the increased infiltration and killing effect of effector T cells. Simultaneously, the levels of immunosuppressive cells and cytokines, such as regulatory T (T_reg_) cells, M2 macrophages, IL-10, and TGF-β, were reduced in the tumor immune microenvironment (TIME). The hydrogel system afforded an immune-friendly revamped TIME and enabled strong (cytotoxic T lymphocyte) CTL killing, leading to efficient chemotherapy-assisted immunotherapy. The restoration of suppressed T cell-mediated immune responses in the TIME without ICBs has also been reported. L-Arginine is an essential amino acid for T-cell function and metabolism [76]. Arginase 1 (ARG1) is known to impair T-cell receptor chain (TCR) synthesis, leading to the inactivation of T-cell responsiveness and decreased efficacy of T-cell therapy [77,78]. Thus, a high drug dose must be continuously delivered for effective ARG1 inhibition. Luan et al. developed an injectable hydrogel composed of a polypeptide block (PLN) and PEG diblock copolymer (PLN-PEG) as a co-delivery platform to release both DOX and the ARG1 inhibitor to the target site in a controlled manner [46]. An ARG1 inhibitor, L-norvaline, was used as an immunomodulating gelator to synthesize PLN-PEG (Figure 2A). A thermoresponsive injectable hydrogel was obtained by this gelator solution, which transformed into a hydrogel within 15 min of injection at the tumor site. The PLN-PEG hydrogel had a porous and interconnected microstructure, with a predominant *β*-sheet conformation. Furthermore, this system guarantees in vitro controlled release of the incorporated L-norvaline in the presence of the proteolytic enzymes in the TME. DOX was also contained within the as-prepared hydrogel solution, resulting in the PLN-PEG@DOX hydrogel. As the hydrogel was continuously degraded, the release of DOX was also efficiently accelerated, leading to the subsequent ICD. With the help of the simultaneously released L-norvaline-blocking ARG1 pathway, this hydrogel system successfully amplified its immunotherapeutic potency (Figure 2B). An in vitro study demonstrated that PLN-PEG@DOX displayed its cytotoxicity and induced ICD in the B16F10 melanoma cells to the same extent as DOX. This hydrogel inhibits the ARG1 pathway, resulting in the reduction in L-arginine catabolism. The urea concentration was measured to analyze the inhibitory effect of PLN-PEG hydrogel on ARG1. Tumor growth was significantly inhibited in both the primary tumor and the abscopal tumor in the B16F10 melanoma mouse model (Figure 2C,D). The population of mature DCs in the PLN-PEG@DOX hydrogel-treated group was remarkably upregulated, more than in the PLN-PEG hydrogel- or DOX-treated groups. Quantitative analysis of various T cells such as the cytotoxic T cells, helper T (T_h_) cells, and T_reg_ cells consistently indicated that the activity and function of T cells were sufficiently restored by the DOX- and ARG1-loaded hydrogel system, with the percentages of CTLs and T_h_ cells being the highest in the primary tumors (both over 12%). In the case of PLN-PEG@DOX, on the other hand, the percentage of T_reg_ cells was the lowest (around 2%) in the same group (Figure 2E). The proportions of CTLs, T_h_ cells, and T_reg_ cells in secondary tumors and splenic lymphocytes were likely to be the same as those in the primary tumors (Figure 2F). Additionally, systemically boosted antitumor immunity ultimately led to the efficient inhibition of pulmonary metastasis. These studies show that combination chemoimmunotherapy using injectable hydrogels provides advanced and effective cancer immunotherapy compared to the conventional monotherapies.

### 3.2. Combinational Photoimmunotherapy

**Figure 3:**
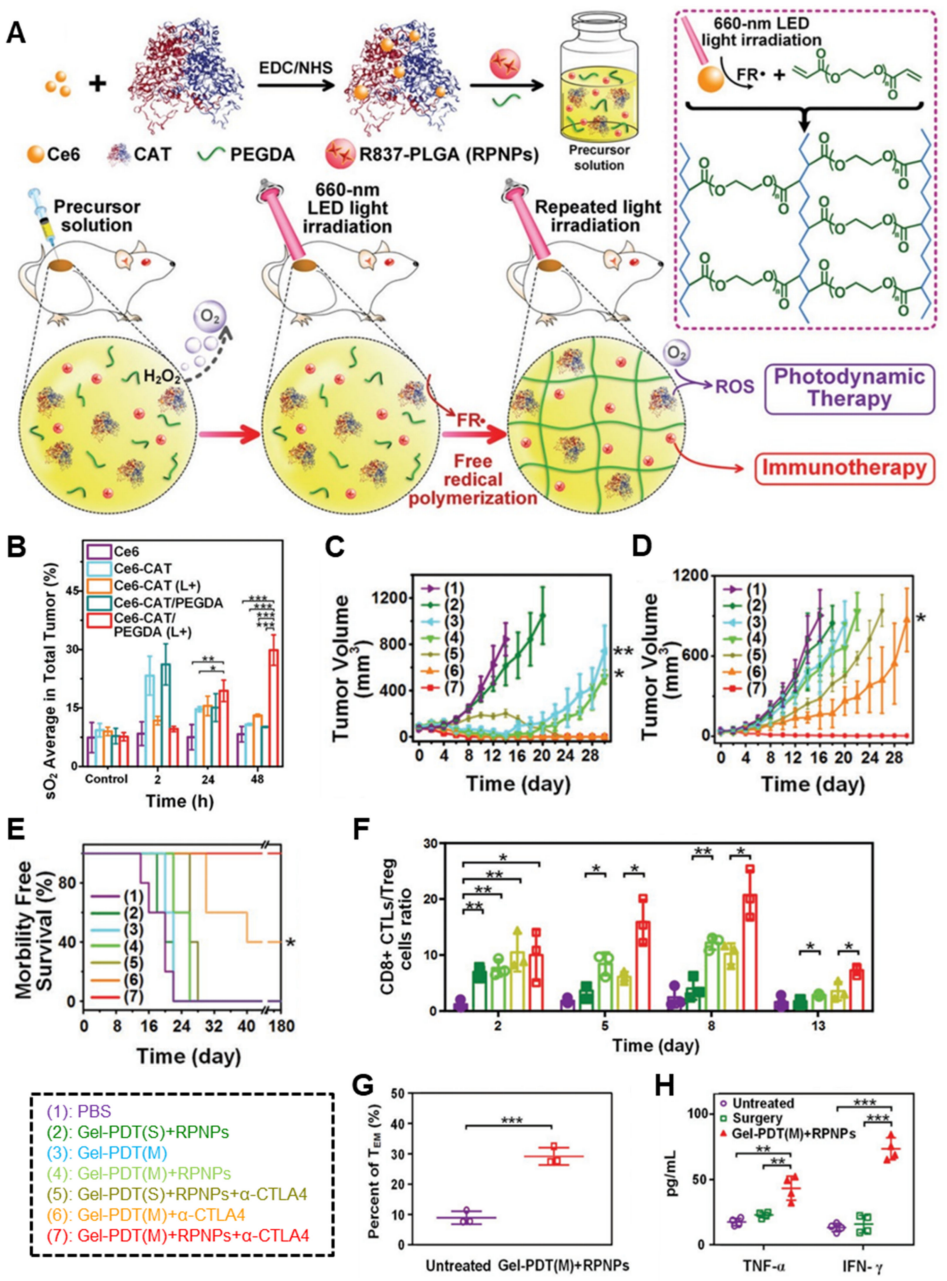
Injectable light-triggered hydrogel with Ce6-CAT and RPNPs for combination photoimmunotherapy. (**A**) Schematic illustration of in situ formation of the light-triggered hydrogel loaded with Ce6-CAT and RPNPs. (**B**) Quantification of levels of the vascular saturated oxygen (sO_2_) in 4T1 tumors by photoacoustic (PA) imaging. Tumor growth curves of the (**C**) irradiated primary tumors and (**D**) non-irradiated secondary tumors after various treatments, including single (S) or multiiple (M) rounds of PDT. (**E**) Survival after treatment. (**F**) CD8^+^ CTL to T_reg_ ratios in secondary tumors. (**G**) Effector memory T (T_EM_) cell proportion in the spleens. (**H**) Quantification of the amount of TNF-α and IFN-γ in sera after the rechallenge. * *p* < 0.05, ** *p* < 0.01, *** *p* < 0.001, one-way ANOVA for indicated comparison. Figures are reproduced from reference [47], with permission from John Wiley & Sons, Inc.

The hydrogel system has made significant contributions that surpass the limits of photodynamic therapy (PDT) and sufficiently induce immune responses, in many cases. For example, antitumor PDT efficacy is limited by the oxygen-deficient hypoxic TME [79]. Liu et al. developed a PEGDA hydrogel loaded with a photosensitizer, an enzyme catalyzing oxygenation, and an immune adjuvant [47]. Specifically, the PDT agent chlorin e6 (Ce6) was conjugated with the catalase (CAT), metabolically providing oxygen to the TME, and this conjugate was then mixed with R837-loaded poly(lactic-*co*-glycolic acid) (PLGA) NPs (RPNPs). Ce6-conjugated CAT is linked via an amide bond, and the natural enzyme activity of CAT was retained. Photoinitiator Ce6 plays a key role in gelation, effectively induced simply by 660 nm light irradiation, producing reactive oxygen species (ROS) and subsequently triggering a free-radical polymerization reaction. This led to the successful hydrogel formation of a network structure with the additional PEGDA and RPNPs (Figure 3A). Application of Ce6-CAT/PEGDA hydrogels results in enhanced Ce6-CAT retention in tumors. After the resultant ICD induction, CTL/T_h_ cell-mediated immunity is boosted and M2 macrophage/T_reg_ cell-mediated activities are suppressed, mainly because of the continuous delivery of R837 by the hydrogel system. The oxygen supply within the TME was increased in this process. Specifically, vascular saturated O_2_ (sO_2_) levels were the highest in the light-irradiated Ce6-CAT/PEGDA group after 24 h. This status displayed a definite trend over time (Figure 3B), implying that Ce6-CAT/PEGDA alleviates tumor hypoxia and simultaneously disrupts the ITM. Enhanced therapeutic effects were observed in terms of tumor growth inhibition and prolonged survival. With the help of the aCTLA-4 checkpoint blockade, the primary tumors were significantly inhibited by multi-round PDT treatment based on the Ce6-CAT/PEGDA hydrogel (Figure 3C). This was mainly due to the robust PDT-induced immune responses, and it appeared that repeated PDT treatment actually enhanced antitumor immunotherapeutic efficacy. With the addition of RPNPs, non-irradiated secondary tumors were also almost entirely eliminated (Figure 3D). The survival time of mice in this group was significantly prolonged (Figure 3E). A much higher percentage of cytotoxic T cells and ratio of CTLs to T_reg_ cells was observed in this group compared with the other groups, regardless of time (Figure 3F). The simultaneous increase in memory T cells and secreted cytokines, such as TNF-α and IFN-γ, indicates that the immune memory effect was successfully achieved (Figure 3G,H), also leading to an obvious abscopal therapeutic effect. From these promising findings, it seems that effective and long-term single-dose cancer immunotherapy could be fully supported by a PDT-based therapeutic approach using an in situ hydrogel platform. A similar hydrogel-based approach using the same agents showed good therapeutic outcomes in the 4T1 breast cancer mouse model [48]. In this study, an alginate (ALG)-based hydrogel loaded with the TLR7 agonist R837 was peritumorally injected, and PDT-based immunotherapy was then performed with the additionally delivered photosensitizer Ce6. PDT is known to trigger systemic antitumor immune responses; nonetheless, PDT-driven antitumor immunity could only be maintained with repeated PDT treatments [80,81]. To overcome this limitation, PDT was driven by the luminescence inherently emitted from persistent luminescence material (PLM) with a broad excitation range. PLM can store the exogenous excitation energy from various light sources, such as ultraviolet (UV), visible, and near-infrared (NIR) light, and even X-rays [82,83,84]. PLM, R837, and additional Ca^2+^ were simply mixed with the ALG solution to synthesize the rechargeable immune hydrogel. The homogeneous structure of this hydrogel was stable, and it helped to homogeneously disperse PLM into the hydrogel. Because of the excellent stability and syringeability of the hydrogel, the in vivo photoactivity of the recharged PL by repeated light-emitting diode (LED) irradiation lasted more than 3 weeks. Both in vitro and in vivo studies indicated that DCs were activated, leading to secretion of immunostimulatory cytokines, such as TNF-α and IL-6. Through the simultaneous increase in CTLs, a reliable antitumor immune effect was achieved by the hydrogel-based PDT-assisted immunotherapy.

**Figure 4:**
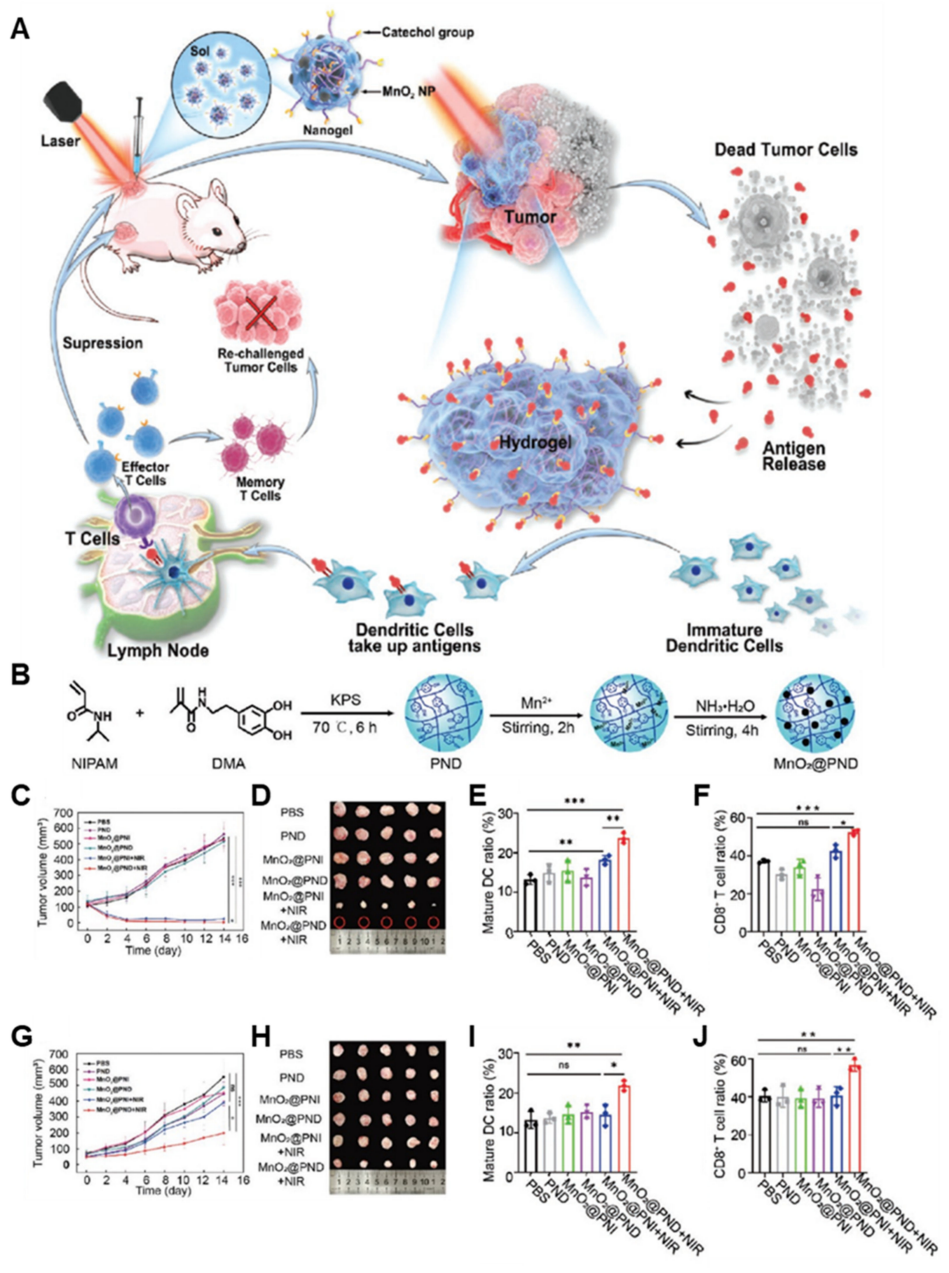
Injectable adhesive hydrogel with MnO_2_ NPs for combination photoimmunotherapy. (**A**) Schematic illustration of the mechanism of the antigen-adhered nanogel with MnO_2_ NPs, eliciting antitumor immune responses. (**B**) Schematic illustration of MnO_2_ NP-loaded hydrogel synthesis. (**C**) Tumor growth curves and (**D**) photographs of primary tumors after treatments. Populations of (**E**) mature DCs in LNs and (**F**) CD8^+^ T cells in primary tumors were analyzed using flow cytometry. (**G**) Tumor growth curves and (**H**) photographs of secondary tumors after treatments. Populations of (**I**) mature DCs in LNs and (**J**) CD8^+^ T cells in secondary tumors analyzed using flow cytometry. * *p* < 0.05, ** *p* < 0.01, *** *p* < 0.001, ns: no significance, one-way ANOVA for indicated comparison. Figures are reproduced from [51], with permission from John Wiley & Sons, Inc.

Photothermal therapy (PTT), meanwhile, is also a promising strategy for cancer therapy. As previously stated, the hydrogel drug delivery system (DDS) has so far made it possible to deliver a high dose of drugs with prolonged release. With the help of the hydrogel DDS, Wang et al. developed a hydrogel DDS using catechol groups and thermosensitive nanogels loaded with NIR photothermal agent (PTA) MnO_2_ for PTT-assisted cancer immunotherapy (Figure 4A) [51]. To obtain MnO_2_ NP-encapsulated poly(*N*-isopropylacrylamide-co-dopamine methacrylamide) (PND) nanogel (MnO_2_@PND), MnCl_2_·H_2_O was added to PND synthesized by the precipitation of the co-polymerization of *N*-isopropylacrylamide (NIPAM) and dopamine methacrylamide (DMA) (Figure 4B). The stronger the hydrophobic interaction, the more nanogels that were formed, with a decrease in the transition temperature. Incorporated MnO_2_ NPs could induce ICD, resulting in high levels of calreticulin (CRT) expression and high mobility group box 1 (HMGB1) release. Strong cytotoxicity and ICD were simultaneously induced in 4T1 cancer cells after intratumoral injection of this hydrogel. The in situ hydrogel specifically captured PTT-induced TAAs, as if it were an antigen reservoir, mainly owing to the strong adhesion-featured catechol loaded in this hydrogel for antigen adsorption. This subsequently enhanced maturation of the recruited immature DCs. The population of mature DCs was remarkably increased in the NIR-irradiated MnO_2_@PND group compared with other groups. The hydrogel platform also exhibited near-perfect in vivo tumor growth inhibition in both primary (Figure 4C,D) and distal tumors (Figure 4G,H) by boosting the innate and adaptive antitumor immune responses in a bilateral 4T1 breast cancer mouse model. In primary tumors, the percentage of mature DCs in lymph nodes (LNs) was up to 23.7% in the case of MnO_2_@PND+NIR (Figure 4E), and the percentage of infiltrated CTLs was up to 52.9% in the same group (Figure 4F). Similarly, the proportion of mature DCs and CTLs was also the highest in the group of MnO_2_@PND+NIR in distal tumors (Figure 4I,J). High levels of TNF-α and IFN-γ were measured in serum from the same group. From these results, it was confirmed that effective antitumor immunity was achieved by MnO_2_@PND hydrogel-based PTT, including a successful abscopal effect. Additionally, cancer relapse was also effectively inhibited, even after consecutive rechallenge, mainly owing to the high level of effector memory T cells. In particular, it was significant that the hydrogel system helped maximize the efficacy of PTT-based cancer therapy and antitumor immunity, with minimal systemic toxicities. PTT-assisted combination immunotherapy resulted in synergistic therapeutic effects, with enhanced coverage and beneficial permeability of tumor cells, similar to other previously discussed PDT-assisted combination immunotherapies [85,86,87]. For example, IR820 is one of the small-molecule organic NIR dyes with good biocompatibility for in vivo fluorescence imaging and PTT of subcutaneous tumors [88]. Lv et al. developed an IR820-loaded PEG-based hydrogel system merged with the nanoparticulated CpG (CpG NPs/IR820 hydrogel) for precise and potent combination cancer immunotherapy [49]. IR820 hydrogel was self-assembled using α-cyclodextrin-conjugated IR820 copolymer and PEG, and it was simply mixed with the PEI-crosslinked CpG. CpG NPs/IR820 hydrogel showed a high storage modulus at 37 °C, and it exhibited strong PTT-induced photothermal cytotoxicity, accompanied by the CpG-mediated immunostimulatory effect of TLR9 activation. Especially in the experimental group treated using the agent-loaded hydrogel along with irradiation, there was a moderate enhancement of DC maturation and CD8^+^ T-cell activation, exhibiting lysis activity, particularly on tumor cells in the draining lymph nodes (DLNs) and spleen, leading to effective tumor growth inhibition in the B16 melanoma mouse model. There was a significant increase in infiltrated B cells that played a crucial role in immunomodulation during tumor progression in the TIME, whereas the infiltrated immunosuppressive cells, such as T_reg_ cells and myeloid-derived suppressor cells (MDSCs) inhibiting the tumoricidal function of CTLs and type 1 helper T cells, were decreased. An immune-friendly TIME was successfully achieved with CpG NPs/IR820 hydrogel-based image-guided combination photoimmunotherapy. Yan et al. tried another combination PTT-immunotherapy approach based on gellan gum (GG) hydrogel loaded with polyoxometalate (POM) and resiquimod (R848) to overcome the current limitations of immunotherapy and conventional PTT, including problems such as toxicity and the lack of long-term efficacy [50]. POM is a PTA that kills tumor cells by converting light to heat, with advantages such as high photothermal conversion efficiency (PTCE), long-term stability, and biodegradability without toxicity [89,90,91]. POM is evenly distributed in the networks of a porous POM@GG hydrogel, with a durable structure and self-healing properties. A subsequent in vitro study indicated the POM clusters rapidly degrade and are stable in the mildly acidic TME, but unstable in ROS-rich conditions. Based on these properties, a synergistic effect of combination therapy was actually exerted by the hydrogel system loaded with the potent modalities, with effective tumor growth inhibition and negligible lung metastases in the 4T1 breast cancer mouse model. Effective tumor killing due to the photothermal effect was attributed to POM, and the subsequently induced ICD-initiated antitumor immune responses were amplified by R848. Immune adjuvant R848 is an agonist of TLR7/8, inducing antitumor immune responses against tumor malignancies [92,93,94]. Strongly induced systemic immunity resulted in the significantly elevated secretion of immunostimulatory cytokines such as TNF-α, IL-2, and IL-6, leading to an antimetastatic effect. Systemic antitumor immunity established by the PTT-immunotherapy resulted in significant inhibition of tumor growth, recurrence, and metastasis, mainly because this hydrogel platform created synergistic effects through complementary modalities.

### 3.3. Combinational Radioimmunotherapy

**Figure 5:**
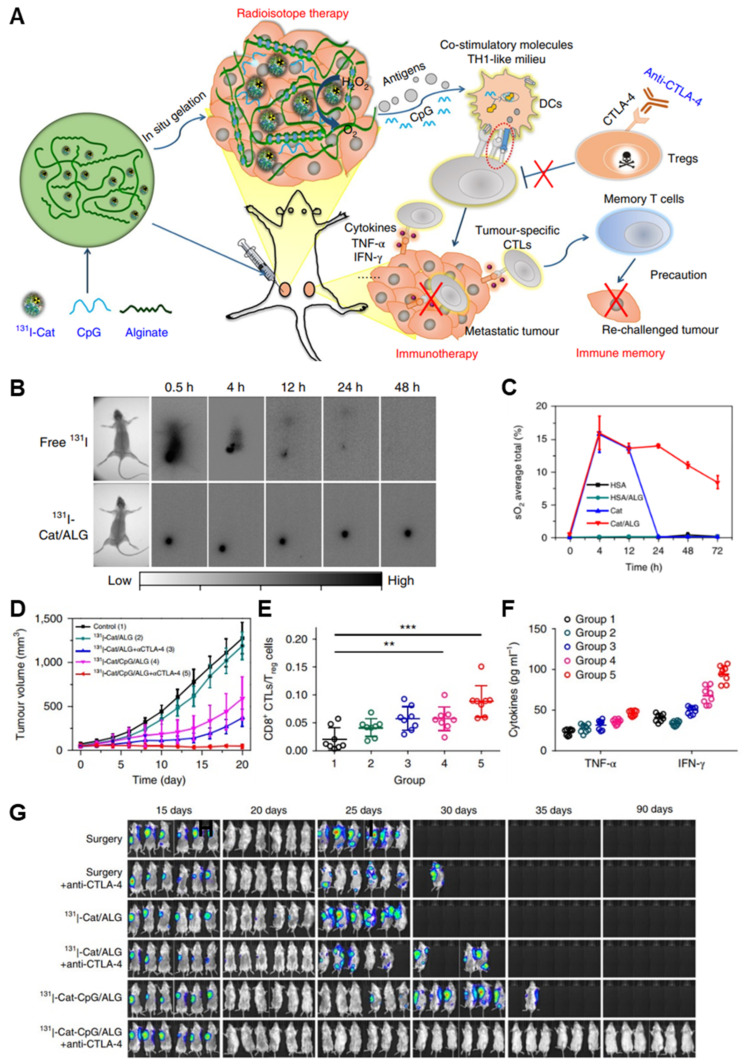
Injectable hybrid hydrogel with ^131^I radioisotope and CpG for combination radioimmunotherapy. (**A**) Schematic illustration of the mechanism of ICB-combined ^131^I- and CpG-loaded hydrogel eliciting antitumor immune responses. (**B**) Gamma scintigraphy whole-body images of ^131^I in 4T1 tumor-bearing mice. (**C**) Quantification of levels of the average total sO_2_ in tumors after treatments. (**D**) Tumor growth curves of primary tumors after treatments. (**E**) CD8^+^ CTL to T_reg_ ratios in secondary tumors. (**F**) Quantification of the amounts of TNF-α and IFN-γ in sera after treatments. (**G**) Visualized bioluminescence image of the implanted FLuc-4T1 tumors representing metastases after treatments. ** *p* < 0.01, *** *p* < 0.001, one-way ANOVA for indicated comparison. Figures are reproduced from [52], with permission from Springer Nature.

**Figure 6:**
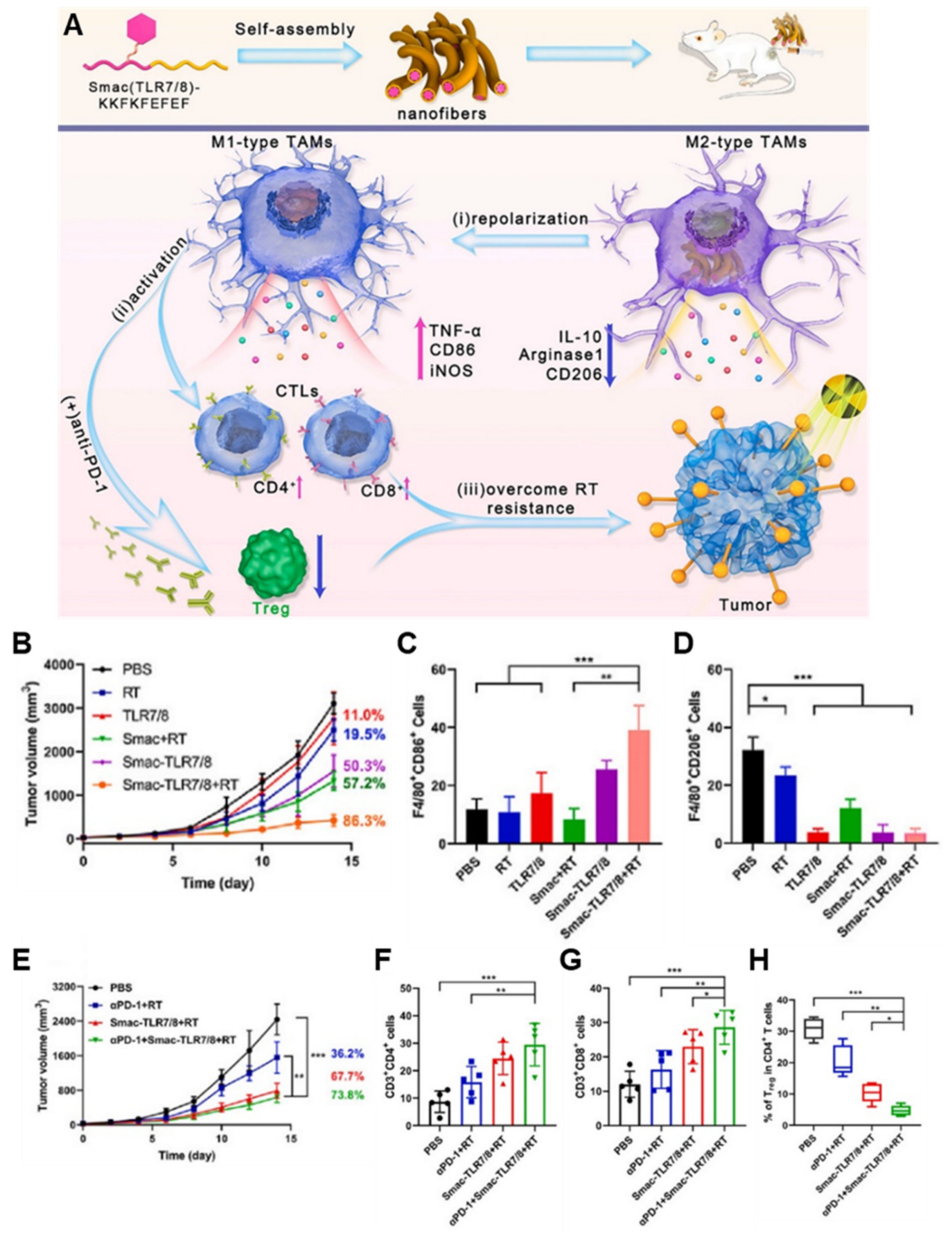
Injectable self-assembled hydrogel with radiosensitive peptide and TLR7/8 agonist for combination radioimmunotherapy. (**A**) Schematic illustration of Smac-TLR7/8 hydrogel overcoming radioresistance by macrophage repolarization. (**B**) Tumor growth curves after treatments. (**C**) M1 and (**D**) M2 macrophage proportions within the tumor tissues quantitatively analyzed by flow cytometry. (**E**) Tumor growth curves after ICB-combined treatments. Populations of (**F**) CD4^+^, (**G**) CD8^+^, and (**H**) regulatory T cells in tumors analyzed using flow cytometry. * *p* < 0.05, ** *p* < 0.01, *** *p* < 0.001, one-way ANOVA for indicated comparison. Figures are reproduced from [54], with permission from Elsevier.

Radiotherapy that exposes cancers to ionizing radiation has been widely used in treatment owing to the immediate and persistent responses, with modest inflammatory changes [95]. Furthermore, the ICD of cancer cells can be induced based on controlling the dose and fractionation, resulting in eliciting anticancer immune responses after radiation [96]. Thus, radiotherapy is a fascinating approach to cancer immunotherapy but has several limitations, such as the off-target toxicity of therapeutic agents and therapeutic ineffectiveness in hypoxic conditions [97]. To overcome these issues, Liu et al. developed a therapeutic ^131^I radioisotope-labeled catalase loaded in alginate (ALG)-based hydrogel (^131^I-Cat/CpG/ALG) to initiate effective radiotherapy, combined with immunotherapy (Figure 5A) [52]. Interestingly, the properties and behavior of the hydrogel were greatly affected by the concentration of ALG solution in the process of gelation. Specifically, the mechanical strength increases with increased ALG concentrations. An in vitro study showed that ALG solutions at concentrations higher than 5 mg/mL quickly transformed into hydrogel with the help of Ca^2+^. This ALG gel did not affect the function of the encapsulated catalase (Cat), but enhanced the stability of the enzyme and even protected a radiolabeled enzyme against protease digestion. ^131^I-Cat/ALG was well retained in the tumor; however, free ^131^I and ^131^I-Cat showed only slight retention and disappeared from the injection site within 48 h (Figure 5B). ^131^I-Cat was retained only slightly longer than free ^131^I, owing to its size. ALG gel helped ^131^I-Cat diffuse more slowly; however, the effect was not sufficient to inhibit the leakage of free ^131^I. Meanwhile, the in vivo oxygenation status in the TME was highly improved over 3 days. The time-dependent average total sO_2_ levels of tumors treated with Cat and Cat/ALG were significantly increased in the first 12 h (Figure 5C). sO_2_ levels of the Cat-treated group dropped drastically after 12 h; on the other hand, the Cat/ALG-treated group retained high levels of sO_2_. This means that Cat was retained stably in ALG and could relieve tumor hypoxia over a long period of time. This contributed to complete tumor elimination during radioisotope therapy (RIT) treatment in the 4T1 breast cancer and patient-derived xenograft (PDX) mouse model. In order to compare the therapeutic efficacy and effectiveness of different treatments, an immunostimulatory cytokine CpG oligonucleotide was co-delivered with hydrogel to trigger strong antitumor immune responses, as previously stated. One of the ICBs, aCTLA-4, was also simultaneously and systemically delivered. In particular, 131I-Cat/CpG/ALG with the aCTLA-4-treated group displayed the most effective tumor inhibition (Figure 5D). This group showed a much higher percentage of CTLs and ratio of CTLs to Treg cells than the other groups (Figure 5E). After these hydrogel-based RIT-assisted combination therapies, CTLs were activated, and the secretion of immunostimulatory cytokines was also increased. The levels of cytokines, such as TNF-α and IFN-γ, which modulate the cytotoxic functions of CTLs, were high in this group (Figure 5F). As with previously introduced successful combination immunotherapies, tumor metastasis was effectively inhibited after treatment with 131I-Cat/CpG/ALG with aCTLA-4 (Figure 5G), and survival was prolonged. An immune memory effect was obviously established based on the increase in effector memory T cells, resulting in the complete prevention of tumor recurrence upon tumor rechallenge. Hybrid hydrogel-based combinations of local radioimmunotherapy and immunostimulatory agents successfully achieved potent therapeutic effects, with systemic antitumor immunity. Recently published studies report on smart hydrogel systems releasing adjuvants with synchronized ICD induced by repeated radiotherapy. For example, Liu et al. designed a combination immunotherapy using an adenosine triphosphate (ATP)-specific aptamer (Aapt)-conjugated ALG-based in situ smart injectable hydrogel [53]. CpG ODNs were also assembled onto this ALG-Aapt, and they were designed to be released in response to the ATP generated by subsequent ICD. Locally applied X-ray irradiation triggered the release of ATP, one of the DAMPs. In response, ALG-based in situ smart hydrogel enabled the release of the reserved CpG hybridized with the Aapt into the TME. Repeated radiotherapy, with additional immune responses, increased the overall therapeutic effects of the combination immunotherapy. Tumor regrowth was prevented, and survival was significantly prolonged. An immune memory effect was achieved simultaneously. In particular, anti-PD-L1 (aPD-L1) was additionally applied to this repeated radiotherapy based on the intratumorally injected hydrogel system. This allowed a potent abscopal effect, and tumor metastasis was also significantly regressed. Another approach to hydrogel-based combination immunotherapy was also used to overcome radioresistance by relieving ITM and enhancing antitumor immunity [54]. In this study, with the help of the TLR7/8 agonist-conjugated radiosensitive peptide hydrogel, tumor-promoting and radio-resistant M2 macrophages were reprogrammed into M1 macrophages through macrophage polarization (Figure 6A) [98,99]. This transparent, porous, and nanofibrous-structured hydrogel was synthesized by self-assembly of the conjugated Smac-TLR7/8 peptide, displaying uniform and compact 3D networks, which are sufficiently beneficial properties for a drug delivery carrier. The pro-apoptosis Smac mimetic peptide was incorporated in the hydrogel to improve the radiosensitivity of the tumor to lower irradiation [100,101]. From the abovementioned characteristics of this hydrogel system, redirection toward tumor-suppressive M1 macrophages was successfully regulated under repeated γ-radiation. Consequently, the secretion of cytokines, such as TNF-α and IFN-γ, was elevated, and antitumor immunity was activated, resulting in effective tumor growth inhibition. Treatment with free TLR7/8 and irradiated Smac hydrogel resulted in 11.0 and 57.2% suppression of the tumor growth rate, respectively. Smac-TLR7/8 hydrogel with radiation displayed the highest tumor inhibition rate (86.3%), owing to the synergistic effect of combination radioimmunotherapy (Figure 6B). In addition, the population of M1 macrophages was significantly higher than that of M2 macrophages in the Smac-TLR7/8 hydrogel in the radiation-treated group (Figure 6C,D). The large number of M1 macrophages secreted pro-inflammation cytokines committed to elite antitumor immune responses. The population of tumor-infiltrating lymphocytes (TILs) was increased; meanwhile, that of Treg cells was decreased. The phenotype changes of the TIME resulted in the consistent enhancement of therapeutic efficacy in the combination immunotherapy. Similarly, promising therapeutic results were also confirmed in the combined approach with anti-PD-1 (aPD-1) in the B16 melanoma (Figure 6E) and 4T1 breast cancer mouse models. The tumor inhibition rate of the aPD-1-combined Smac-TLR7/8 hydrogel in the radiation-treated group was 11% higher than in the non-combined therapy group. This combined therapy group exhibited a higher population of Th cells and CTLs (Figure 6F,G) and a lower population of Treg cells (Figure 6H). Significantly, this study demonstrates that the hydrogel DDS-based radiotherapy-assisted immunotherapy is effective enough to elicit antitumor immunity and overcome radioresistance caused by TAM repolarization.

## 4. Conclusions and Perspectives

In this review, we summarized recent advances in injectable hydrogels for combination cancer immunotherapy. Based on advances in materials chemistry, polymer physics, and fabrication strategies, injectable hydrogels provide various advantages in cancer treatment, including in cancer immunotherapy. Because of exclusive advantages, such as biocompatibility, biodegradability, easy encapsulation, and local sustained release of immunotherapeutics, injectable hydrogels have shown the potential to overcome the limited therapeutic efficacy of systemically administered immunotherapeutics and their associated systemic toxicity. Furthermore, injectable hydrogels can elicit anticancer immune responses through the combination of one or more immunotherapeutics, as well as therapeutic modalities, significantly inhibiting the progression and metastasis of cancer. However, the heterogeneous and suppressed TIME of individual cancer patients is often a major factor in the failure of cancer immunotherapy. Thus, innovative and optimized designs of injectable hydrogels are needed to achieve ideal cancer immunotherapy based on the understanding of the immune system and TIME. For example, an acidic TME increases the engagement between the T-cell immunoreceptor and the Ig and ITIM domains (TIGIT) and CD155, restricting T-cell responses. In this regard, designing an extracellular pH-modulating injectable hydrogel to neutralize tumor acidity may help to impair CD8+ T-cell activity, leading to the improved therapeutic efficacy of immune checkpoint therapy. Meanwhile, injectable hydrogels can be designed not only to deliver therapeutic agents, but also to stimulate anticancer immune responses. Designing immunogenic properties into the decomposition products of injectable hydrogels can continuously stimulate immune responses by self- or external stimuli-based degradation. Finally, with increased efforts toward optimizing their physicochemical properties and biosafety and developing strategies to combine immunotherapy using therapeutic modalities, injectable hydrogels have great potential to accelerate clinical translation with significant therapeutic efficacy.

## Figures and Tables

**Table 1:** Summary of injectable hydrogels used for combination cancer immunotherapy. Abbreviations—A_apt_: ATP-specific aptamer Cat: catalase; α-CD: α-cyclodextrin; DOX: doxorubicin; GG: gellan gum; PDX: patient-derived xenografts; PEG: polyethylene glycol; PEGDA: polyethylene glycol double acrylate; PEGSH: polyethylene glycol thiol; PLN: polypeptide block; PND: poly(*N*-isopropylacrylamide-co-dopamine methacrylamide); POM: polyoxometalate; TLR: toll-like receptor; HAMA: hyaluronic acid methacrylate; UPyMA: 2-(3-(6-methyl-4-oxo-1,4-dihydropyrimidin-2-yl)ureido)ethyl methacrylate; DEGMA: diethylene glycol methacrylate; PEI: polyethyleneimine; CA: *cis*-aconitic anhydride; DCs: dendritic cells; PLGA NPs: poly(lactic-co-glycolic acid) nanoparticles; PLM: persistent luminescence material (Zn_1.25_Ga_1.5_Ge_0.25_O_4_:0.5%Cr^3+^, 2.5%Yb^3+^, 0.25%Er^3+^).

Types	Hydrogels	Therapeutic Agents	Target Cancers	Strategies	Ref.
Chemoimmunotherapy	Melittin conjugated RADA_32_	Melittin, DOX	B16F10 melanoma	- Chemoimmunotherapy using self-assembling peptide hydrogels that can induce cell membrane disruption in combination with DOX for eliciting antitumor immune responses. - Remodeling ITM by regulating innate immune cells and depleting M2-like TAMs. - Synergistic inhibition effect by disrupting tumor cell membrane associated with MDR.	[42]
	PEGDA, PEGSH	DOX, R837	B16F10 melanoma	- Chemoimmunotherapy using DOX- and immune adjuvant R837-loaded in situ crosslinkable hydrogel composed of four-arm PEGSH and PEGDA. - Synergistic therapeutic effect of DOX- and R837 on the metastatic progression of melanoma.	[43]
	HAMA, DEGMA, UPyMA	DOX, PD-L1 antagonist D peptide	CT26 colorectal carcinoma	- DEGMA and UPyMA-conjugated HAMA supramolecular hydrogel encapsulating DOX and PD-L1 antagonist D peptide. - PD-L1 antagonist D peptide-based locoregional block of the PD-1/PD-L1 pathway, resulting in potentiating T cell-mediated immune responses and minimizing side effects.	[44]
	PEG-4000, α-CD	PEI-CA-DOX, CpG, DCs	B16 melanoma	- pH-sensitive adjuvant DOX prodrug- and DCs co-laden hydrogel for alleviating immunosuppressive TME by increasing tumor antigen generation and antigen presentation. - Promoting antigen presentation by the adjuvant and the implanted exogenous DCs.	[45]
	PLN-PEG	DOX, L-norvaline	B16F10 melanoma	- DOX-loaded L-norvaline-based polypeptide-*b*-PEG (PLN-PEG) hydrogel for combination chemoimmunotherapy by inducing ICD and blocking the arginase 1 (ARG1) pathway. - High drug loading efficiency and negligible side effects by using ARG1 inhibitor L-norvaline.	[46]
Photoimmunotherapy	PEGDA	Ce6-Cat, R837-loaded PLGA NPs	4T1 mammary carcinoma and CT26 colorectal carcinoma	- Ce6-CAT- and R837-loaded PLGA NPs-co-laden light-triggered hydrogel for reversing immunosuppressive TME in photodynamic immunotherapy of cancers. - Effective multi-round PDT and immunotherapy based on persistent tumor hypoxia relief.	[47]
	Alginate-Ca^2+^	Ce6, R837	4T1 mammary carcinoma	- “Turning solid into gel” strategy using PLM- and R837-co-laden alginate-Ca^2+^ hydrogel for rechargeable photodynamic immunotherapy of cancers. - Sustained and rechargeable efficient PDT with the PLM-based continuous internal light source.	[48]
	PEG, IR-820-α-CD	IR820, CpG	B16 melanoma	- Genipin-crosslinked CpG NPs-loaded PEG-IR-820-α-CD hydrogel for combinational photothermal immunotherapy. The hydrogel induces a hyperthermal effect, generating tumor antigens. - High loading efficiency and long-term release of CpG by self-crosslinked structures. - Image-guided therapy based on the PEG-IR-820-α-CD hydrogel for precise tumor treatment.	[49]
	GG	POM, R848	4T1 mammary carcinoma	- Dawson-type (P_2_Mo_18_) POM- and R848-co-laden gellan gum (GG) hydrogel for combinational photothermal immunotherapy. - High photothermal conversion effect and photostability during repeated treatment cycles.	[50]
	PND	MnO_2_	4T1 mammary carcinoma	- MnO_2_ nanoparticle-based in situ-formable thermosensitive nanogels that can capture tumor antigen for enhancing anticancer immune response and immune memory effect after photothermal immunotherapy. - Tumor antigen adsorption by using the adhesive antigen reservoir system, resulting in prolonging immune stimulation and enhancing DC recruitment.	[51]
Radioimmunotherapy	Alginate-Ca^2+^	^131^I-Cat, CpG	4T1 mammary carcinoma and CT26 colorectal carcinoma, prostate cancer PDX model, rabbit VX2 liver cancer	- ^131^I-Cat- and CpG-co-laden injectable hydrogel that causes oxygenation by Cat-triggered decomposition of endogenous H_2_O_2_, enhancing radioimmunotherapy efficacy with a low dose of radioactivity. Based on the “vaccine-like” function of hydrogels, distant metastatic cancers were effectively eliminated when combining treatment with anti-CTLA-4 antibody. - Less invasive and easy application compared to the implantable ^125^I beads. - Long-term relief of tumor hypoxia and effective primary tumor elimination under low doses of radiation.	[52]
	A_apt_-conjugated alginate	CpG-cAptamer, oxaliplatin	4T1 mammary carcinoma and CT26 colorectal carcinoma	- A_apt_-conjugated alginate-based injectable hydrogel that can hybridize with CpG-cAptamer, resulting in ATP-responsive release of CpG by low doses of oxaliplatin or X-rays for the synergistic immune responses. - Immune adjuvant release synchronized with low-dose repeated RT, resulting in synergistic tumor elimination and long-term immune-memory effects.	[53]
	Smac-conjugated TLR7/8 receptor agonist peptide	Smac-conjugated TLR7/8 receptor agonist peptide	B16 melanoma	- Self-assembled Smac-TLR7/8 peptide hydrogel that can repolarize tumor-associated macrophages into M1 type for improving anticancer immune responses by relieving radio-resistant TME. - Modulating the ITM and overcoming the radioresistance by reprogramming TAMs polarization toward the M1 phenotype. - Improving radio sensitivity and reducing the radiation dose due to the Smac mimetic peptide.	[54]
